# Renal abscess after the Fontan procedure: a case report

**DOI:** 10.1186/1752-1947-5-50

**Published:** 2011-02-04

**Authors:** Anurag Mehrotra, Pallavi Khanna, Suresh Kumar, Georgi Abraham

**Affiliations:** 1 Department of Nephrology, Madras Medical Mission, Chennai, India; 2 Department of Pediatric Cardiology, Madras Medical Mission, Chennai, India

## Abstract

**Introduction:**

The Fontan procedure is an intervention that helps to correct single ventricle physiology. There are many known long-term complications of 'Fontan physiology'. However, the occurrence of renal abscess in such patients has not yet been reported in the literature. The first generation of adults has now undergone the procedure and it is necessary to be aware of the long-term outcomes and complications associated with it.

**Case presentation:**

We report the case of a 22-year-old South Indian man who had developed a staphylococcal renal abscess against a background of xanthogranulomatous pyelonephritis, nine years after Fontan surgery. He presented to our hospital with a high-grade fever of 25-days duration but with no other symptoms. Physical examination identified costovertebral angle tenderness and pedal edema. An ultrasound scan revealed a mass in his left kidney. The results of a computed tomography scan were consistent with a renal abscess. Despite treatment with the appropriate parenteral antibiotics, there was no change in the size of the abscess and a left nephrectomy was performed as a curative procedure.

**Conclusions:**

The learning points here are manifold. It is important to be aware of the possibility of renal abscess in a post-procedural patient. The early diagnosis of a septic focus in the kidneymay help to prevent the rare outcome of nephrectomy.

## Introduction

Fontan surgery is a form of definitive palliation. It was first described in 1971 by Fontan and Baudet as a procedure for "physiological pulmonary blood flow restoration, with suppression of right and left blood mixing" [1].

Better, and later, hemodynamic modifications include the extracardiac and fenestrated Fontan procedure, which is indicated for tricuspid atresia, hypoplastic left heart syndrome, double inlet ventricle and isomerism [2]. We describe the case of a man with double outlet right ventricle and severe pulmonary stenosis who underwent a fenestrated Fontan procedure at the age of 13. He developed a left renal abscess nine years after the procedure. The occurrence of a renal abscess in a patient who has undergone the Fontan procedure has not been previously reported in the literature.

## Case presentation

A 22-year-old South Indian man with a previous history of Fontan surgery at the age of 13 for double outlet right ventricle with severe pulmonary stenosis and straddling tricuspid valve presented with a spiking high-grade fever of 25-day duration. He had no history of cough, ear discharge, respiratory infection, dysuria, diarrhea, gastrointestinal distress or vomiting.

His past history included surgery for a brain abscess at the age of 13, Fontan surgery at the age of 13, ocular surgery for retinal detachment at the age of 16, and multiple small skin abscesses chiefly on his left foot, which recurred after treatment and led to an excision of an abscess on his foot. At the age of 20, he was diagnosed with protein-losing enteropathy.

The last echocardiography performed before his hospitalization showed a right to left flow in the Fontan circuit, signifying a flow of de-oxygenated blood from the intended pulmonic to the systemic circulation.

On physical examination, he was found to be febrile with a temperature of 39°C on admission, a pulse rate of 88 per minute, a respiratory rate of 26 per minute, blood pressure of 98/60 mmHg, and oxygen saturation of 89 percent in room air. A head to toe examination identified clubbing of his nails, a median sternotomy scar, mild abdominal distension and pedal edema. His teeth and oral cavity were found to be normal. He was 174 cm tall and weighed 51 kg.

His laboratory data on admission showed the following: white blood cells (WBCs) 14,000/mm^3^, neutrophils 84.5 percent, lymphocytes 8.3 percent, eosinophils 0.2 percent, erythrocyte sedimentation rate 45 mm/hour, hemoglobin 10.1 g/dL, urea 19 mg/dL, serum creatinine 0.6 mg/dL, sodium 122 mmol/L, potassium 3.7 mmol/L, total protein 3.7 g/dL, serum albumin 1.3 g/dL and serum globulin 2.4 g/dL. He tested negative for hepatitis B surface antigen, hepatitis C virus and human immunodeficiency virus. A further work-up for immune deficiency could not be performed for logistical reasons. A urine analysis showed 20-25 red blood cells and 10-12 WBCs per high-power field.

**Figure 1:**
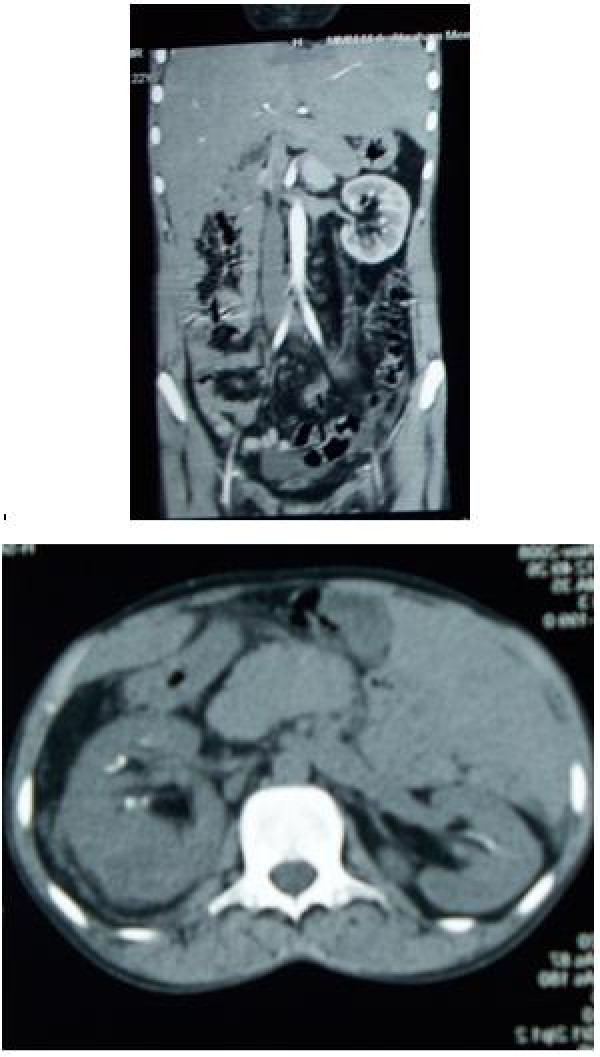
**Longitudinal and horizontal abdominal computed tomography images of the affected kidney**.

A 2 D echocardiography revealed no vegetations. An ultrasound scan revealed a mass in his left kidney measuring 7.2 × 4 cm. A computed tomography (CT) scan showed a hypodense area in the lower pole of his left kidney measuring 5.28 × 6.22 cm, consistent with a renal abscess, which was percutaneously aspirated and grew highly sensitive *Staphylococcus aureus*. Special staining for acid-fast bacilli was negative. Figure 1 shows the CT images. His blood cultures were repeatedly negative. One of the urine cultures grew *Escherichia coli *and *Enterococcus *species. The *E. coli *was sensitive to amikacin, cefoperazone and/or sulbactam, gentamicin, imipenem, meropenem, natamycin, nitrofurantoin, and piperacillin and/or tazobactam. The *Enterococcus *species was sensitive to amoxicillin and clavulanic acid, gentamicin, imipenem, linezolid, meropenem and nitrofurantoin.

On the basis of the sensitivity of the *S. aureus *isolated from the abscess, he was treated with intravenous gentamicin, 80 mg at eight-hourly intervals, and with intravenous teicoplanin, 400 mg once per day.

**Figure 2:**
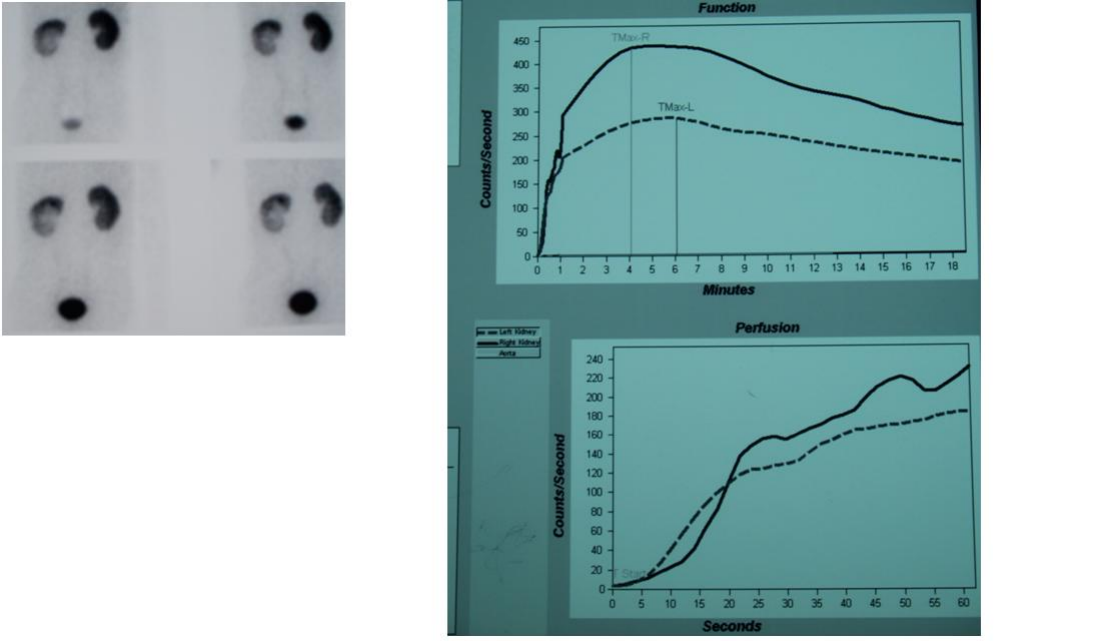
**Technetium-99 m renogram images of the affected kidney**.

He continued experiencing spikes of high-grade fever, and a repeat ultrasound after 12 days of appropriate therapy showed only minimal resolution of the lesion. Surgery was anticipated. A technetium-99 m renogram was performed to see the split function of the kidney with the abscess and to determine whether or not a partial nephrectomy could be performed. The renogram revealed a total glomerular filtration rate of 94 mL/min, with the left kidney contributing 36 mL/min and the right kidney 58 mL/min, and no evidence of obstruction. Figure 2 shows the results of the technetium-99 m renogram.

**Figure 3:**
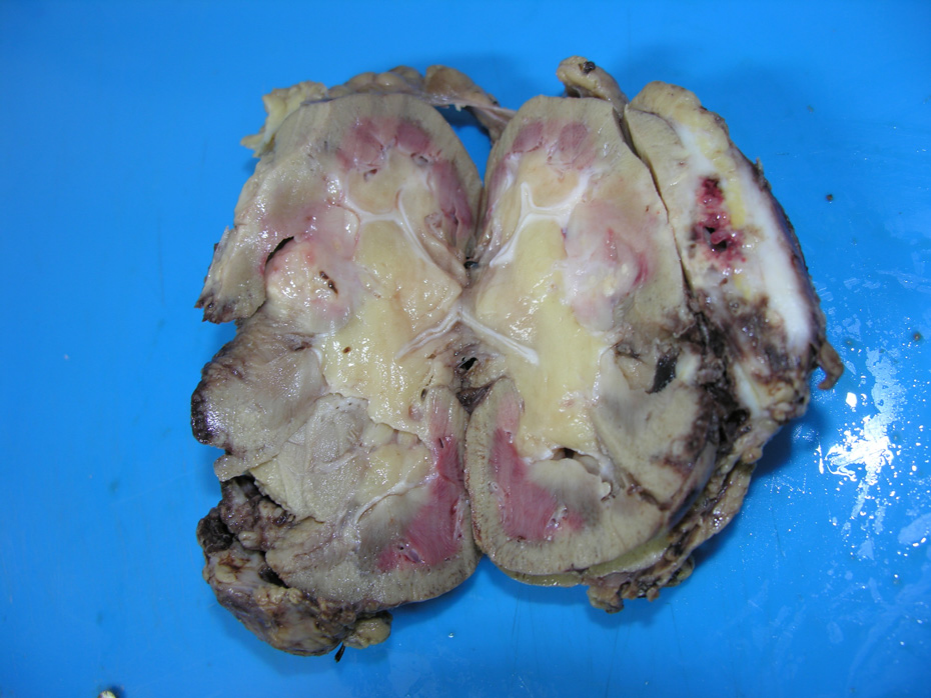
**Gross specimen of the affected kidney**.

**Figure 4:**
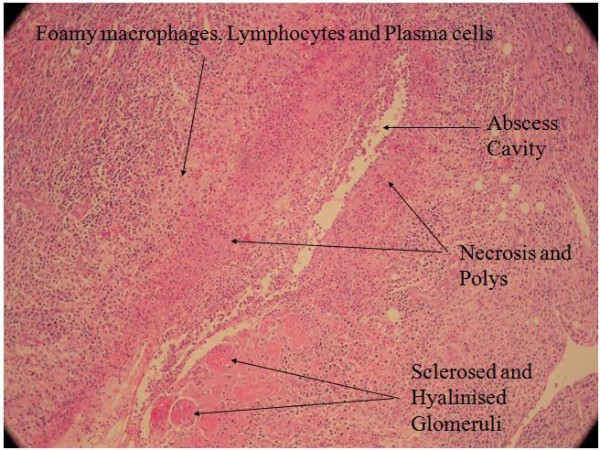
**Labelled histopathological picture of the affected kidney**.

**Figure 5:**
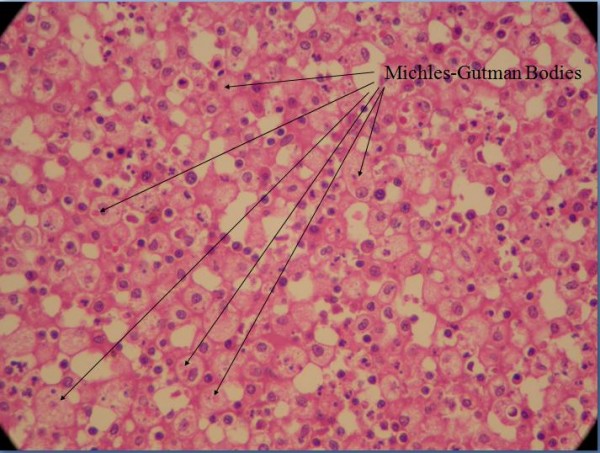
**Labelled histopathological picture of the affected kidney**.

In view of the persistence of the abscess, he underwent a surgical exploration of the renal bed. An attempt was made to carry out a partial nephrectomy of the affected region. However, this failed and so a left nephrectomy was performed. Figure 3 shows the nephrectomy specimen. Figures 4 and 5 show the histopathological picture.

Post-operatively, his fever subsided and the antibiotic coverage was continued for one week with teicoplanin and gentamicin. At the time of his discharge, his serum creatinine level was 1.1 mg/dL.

A histopathological examination of the diseased kidney revealed infiltrates of lymphocytes, plasma cells and histiocytes. The replacement of renal parenchymal tissue by sheets of foamy histiocytes admixed with neutrophils was observed and this was consistent with xanthogranulomatous pyelonephritis. A special stain for acid-fast bacilli was negative. Clinical, radiological and histopathological examinations failed to provide any evidence of an obstructive lesion in his urinary tract or of renal calculi.

Two weeks after his discharge from hospital, he complained of fever. A CT scan of his abdomen was performed and a residual renal bed abscess was found. A pigtail catheter was inserted and daily aspiration and antibiotic instillation were performed. A week later he was discharged again, with oral antibiotics.

About seven months after the surgery, he remained in a perfect state of health without reports of further infection. This also signified the absence of inherent immune deficiency.

## Discussion

Renal abscess is defined as the presence of suppurative material in either the Gerota's fascia or within the kidney, which may be perinephric, renal cortical or corticomedullary [3]. Predisposing factors to this condition include diabetes, renal stone disease, ureteral obstruction, immunosuppression, chronic urinary retention and urological intervention [4].

The current predominant microbiological flora in renal abscesses are Gram-negative organisms, with *E. coli *being isolated from 26.5 percent of cases. The most common Gram-positive organism is *S. aureus*, as seen in 18.3 percent of cases. Abscesses caused by *S. aureus *are believed to result either from bacteremia produced by infection at another site or as a result of immunosuppression. As has previously been reported, a staphylococcus renal abscess had concomitant cutaneous lesions in one of our patients. It has also been reported that organisms isolated from a urine culture parallel the bacteriology of the abscesses; however, this is not true in 6.6 percent of cases [4]. The results of urine and blood cultures are positive in fewer than 50 percent of patients with a renal abscess [4].

As indicated in the literature, the diagnosis was also difficult in our case report; he presented with only fever and costovertebral angle tenderness. He had no stigmata of infective endocarditis, except clubbing of the nails [4].

An immunocompromized state is a predisposing factor accounting for up to 4.6 percent of cases, as seen in a recent review [4]. Given our patient's history of brain abscess, palliative cardiac surgery, recurrent staphylococcal skin abscesses, protein-losing enteropathy and a low lymphocyte count, it was likely that he might have been immunocompromized. Adult patients with congenital heart disease have elevated levels of inflammatory cytokines and bacterial endotoxins, which contribute to the impairment of their immune system [5].

Fontan surgery is a generic name for surgical procedures connecting the systemic venous circulation to the pulmonary circuit in a patient with a single ventricle physiology, in an effort to restore saturation. As in our case report, patients who have undergone the Fontan procedure typically live to adulthood; in a series of 180 patients only two died of sepsis [6]. To the best of our knowledge, there have been no case reports of renal abscess following this procedure.

Though the renal abscess was found nine years after the procedure, the lack of urinary symptoms and the lack of any immunodeficiency or infection with *S. aureus *led us to believe that this renal abscess was not a primary event. We attribute it to the altered hemodynamics of a long-standing Fontan circuit. The abnormal pressure-volume relationships, frequent adaptive changes in the ventricles from being overloaded to "overgrown" after the procedure, chronic hypoxemia, ventricular dysfunction and residual shunts might have been responsible for the abscess [7].

There was no evidence of infective endocarditis on echocardiography and his blood cultures were repeatedly negative. Most perinephric abscesses are treated by interventional treatment: surgical drainage (24 percent), percutaneous drainage (42 percent), or nephrectomy (24 percent), along with appropriate antibiotic therapy, as in our case report [4]. We attempted percutaneous drainage under ultrasound guidance in our case report, but this was not successful. Consequently he underwent a surgical exploration of the renal bed. An anatomical examination at the time of the surgical exploration provided evidence of the extent of the process and, as a result, a nephrectomy was performed. Nephrectomy is usually reserved for non-functioning kidneys secondary to nephrolithiasis.

To our surprise, the histological features were suggestive of xanthogranulomatous pyelonephritis, suggesting a protracted infective process. This is a special form of pyelonephritis characterized by chronicity and the presence of foamy cells, islands of abscesses and granulomas. Most patients with this histopathological nature of pyelonephritis initially present with non-specific features such as fever of unknown origin, anorexia, nausea, weight loss, malaise and constipation, typically delaying the diagnosis by three months to nine years after the initial presentation [8].

## Conclusions

The Fontan procedure is a complicated surgical endeavor which aims to correct a highly aberrant physiology. The procedure has long-term complications which have been previously reported. Repeated episodes of septic foci and, as in our case report, a renal abscess after the peri-operative period have not previously been reported. To the best of our knowledge, this is the first such case report.

As the long-term consequences of Fontan circuit are a subject of study, physicians should be reminded of the possibility of an unknown foci of sepsis such as a renal abscess.

## Abbreviations

CT: computed tomography; dL: decilitre; mg: milligram; mm: millimetre; WBC: white blood cells.

## Consent

Written informed consent was obtained from the patient for publication of this case report and any accompanying images. A copy of the written consent is available for review by the Editor-in-Chief of this journal.

## Competing interests

The authors declare that they have no competing interests.

## Authors' contributions

AM and PK analyzed and interpreted the patient data regarding the renal disease. SK was our patient's primary cardiologist and made major contributions to the manuscript. GA was our patient's nephrologist. Both GA and SK were involved in clinical decision-making in this case. The manuscript was prepared by AM under the supervision of GA. All authors read and approved the final manuscript.
